# Supplementary material for: Quantitative H2S-mediated protein sulfhydration reveals metabolic reprogramming during the integrated stress response
Source: eLife. 2015 Nov 23;4:e10067. doi: 10.7554/eLife.10067 (PMC4733038; doi:10.7554/eLife.10067)
Supplement: Supplementary file 1. — PAG was added for the last 3.5 hr of Tg-treatment in the indicated experimental samples. Notes: the reported serine M+2 labeling reflects the two carbon atoms in the GC-MS fragment ion that was quantitated, and is expected to be predominantly derived from fully-labeled (M+3) serine. DOI: http://dx.doi.org/10.7554/eLife.10067.036 [file elife-10067-supp1.docx]

| **Glucose flux** | **GLU-->G3P** | **GLU-->Glycerol** | **GLU-->3PG** | **GLU-->Lac** | **GLU-->ALA** |
| --- | --- | --- | --- | --- | --- |
| Control | 5.49±0.13 | 5.08±1.91 | 59.46±1.27 | 48.52±4.76 | 67.84±1.92 |
| Tg | 2.76±0.07** | 3.36±0.28 | 71.77±2.09** | 75.34±3.68** | 83.78±1.93** |
| Tg+PAG | 1.74±0.14** | 2.17±0.15** | 62.91±2.27* | 65.95±2.01* | 46.02±2.59** |

GLU: glucose, G3P: glycerol-3-phosphate, 3PG: 3-phosphoglycerate, Lac: lactate, ALA: alanine. *p<0.05**, *p<0.*01**.

| **TCA flux** | **Citrate/acetylCoA** | **Citrate/OAA** | **Succinate** | **Fumarate** | **Malate** |
| --- | --- | --- | --- | --- | --- |
| Control | 42.65±1.08 | 48.59±0.86 | 16.39±2.73 | 25.03±0.66 | 27.73±0.43 |
| Tg | 33.65±0.15** | 38.14±0.27* | 13.26±0.57 | 22.08±0.18** | 13.69±0.72** |
| Tg+PAG | 43.24±0.38** | 51.98±0.68* | 19.93±1.12** | 35.23±0.44** | 31.37±0.32** |

Citrate/acetylCoA: acetylCoA moiety of citrate, Citrate/OAA: oxaloacetate moiety of citrate. *p<0.05**, *p<0.*01**.

| **AA flux** | **GLU-->Glycine** | **GLU-->Serine** | **GLU--> Proline** | **GLU--> Aspartate** | **GLU-->Glutamine** |
| --- | --- | --- | --- | --- | --- |
| Control | 32.90±1.40 | 5.24±0.25 | 6.55±0.17 | 12.04±0.14 | 11.94±0.26 |
| Tg | 36.99±2.54 | 1.72±0.10** | 5.82±0.09** | 8.68±0.06** | 8.86±0.13** |
| Tg+PAG | 21.20±0.79** | 1.88±0.13 | 12.31±0.04** | 13.33±0.07** | 15.90±0.12** |

AA: amino acid, GLU: glucose. *p<0.05**, *p<0.*01**.

| **Concentration** | **G3P** | **Glycerol** | **3PG** | **Lactate** | **Alanine** | **Valine** |
| --- | --- | --- | --- | --- | --- | --- |
| Control | 0.046±0.001 | 0.091±0.003 | 0.673±0.104 | 0.556±0.044 | 1.461±0.110 | 0.224±0.014 |
| Tg | 0.112±0.013** | 0.086±0.011 | 0.756±0.104 | 0.364±0.043* | 2.032±0.193* | 0.170±0.014* |
| Tg+PAG | 0.078±0.008 | 0.074±0.006 | 0.545±0.055 | 0.443±0.018 | 0.410±0.025** | 0.166±0.010 |

Relative concentrations of glycolysis metabolites. GLU: glucose, G3P: glycerol-3-phosphate, 3PG: 3-phosphoglycerate, Lac: lactate, ALA: alanine *p<0.05**, *p<0.*01**

| **Concentration** | **Citrate** | **Succinate** | **Fumarate** | **Malate** |
| --- | --- | --- | --- | --- |
| Control | 0.016±0.002 | 7.609±1.611 | 0.030±0.002 | 0.048±0.002 |
| Tg | 0.017±0.002 | 4.993±0.514 | 0.047±0.006* | 0.084±0.008** |
| Tg+PAG | 0.011±0.001* | 4.738±0.870 | 0.017±0.001** | 0.026±0.003** |

Relative concentrations of TCA metabolites. *p<0.05**, *p<0.*01**.

| **Concentration** | **Glycine** | **Serine** | **Proline** | **Aspartate** | **Glutamine** |
| --- | --- | --- | --- | --- | --- |
| Control | 2.17±0.19 | 0.14±0.01 | 2.01±0.10 | 3.84±0.18 | 0.53±0.01 |
| Tg | 2.51±0.18 | 0.33±0.03** | 2.19±0.22 | 2.66±0.29* | 0.66±0.10 |
| Tg+PAG | 2.66±0.12 | 0.19±0.01** | 1.63±0.11 | 2.81±0.14 | 0.75±0.07 |

Relative concentrations of amino acids *p<0.05**, *p<0.*01**.
